# Influence of hydrothermal carbonization conditions on the porosity, functionality, and sorption properties of microalgae hydrochars

**DOI:** 10.1038/s41598-023-35331-0

**Published:** 2023-05-26

**Authors:** Ivan Kozyatnyk, Veronica Benavente, Eva Weidemann, Francesco G. Gentili, Stina Jansson

**Affiliations:** 1 Department of Chemistry, Umeå University, 901 87 Umeå, Sweden; 2 Department of Forest Biomaterials and Technology, Swedish University of Agricultural Sciences, 901 83 Umeå, Sweden; 3 Present Address: Department of Health, Medicine and Caring Sciences, Unit of Clinical Medicine, Occupational and Environmental Medicine, Linköping University, 581 83 Linköping, Sweden

**Keywords:** Environmental chemistry, Green chemistry

## Abstract

Green microalgae is a possible feedstock for the production of biofuels, chemicals, food/feed, and medical products. Large-scale microalgae production requires large quantities of water and nutrients, directing the attention to wastewater as a cultivation medium. Wastewater-cultivated microalgae could via wet thermochemical conversion be valorised into products for e.g., water treatment. In this study, hydrothermal carbonization was used to process microalgae polycultures grown in municipal wastewater. The objective was to perform a systematic examination of how carbonization temperature, residence time, and initial pH affected solid yield, composition, and properties. Carbonization temperature, time and initial pH all had statistically significant effects on hydrochar properties, with temperature having the most pronounced effect; the surface area increased from 8.5 to 43.6 m^2^ g^−1^ as temperature was increased from 180 to 260 °C. However, hydrochars produced at low temperature and initially neutral pH generally had the highest capacity for methylene blue adsorption. DRIFTS analysis of the hydrochar revealed that the pH conditions changed the functional group composition, implying that adsorption was electrostatic interactions driven. This study concludes that un-activated hydrochars from wastewater grown microalgae produced at relatively low hydrothermal carbonization temperatures adsorb methylene blue, despite having low surface area.

## Introduction

Green microalgae exhibit high photosynthetic efficiency and a fast growth rate, which in combination with their carbon-capturing capability and possibility for cultivation on non-arable land^1–3^ makes them a potential feedstock for biofuel, biochemicals, food/feed, or medical production^4–6^. Microalgal biomass production requires large quantities of water and nutrients, which has generated interest in using wastewater as a cultivation medium^7^. Microalgae have been shown to recover 82–92% of nitrogen and 58–98% of phosphorus present in wastewater while reducing its chemical oxygen demand by up to 62%^8,9^. Wastewater microalgae cultivation also requires a carbon dioxide source, and using incineration flue gases for this purpose increases the societal and environmental benefits of the approach by capturing some of the CO_2_ released during incineration and thus helping mitigate climate change^10,11^. While most microalgae cultivation facilities are located in temperate or tropical climate zones, studies have shown that using locally sourced strains of microalgae enables wastewater reclamation and biomass generation even in nearly subarctic climate zones^12^, which have short summer seasons, albeit with long daylight hours, and comparatively low average temperatures.

A challenge of using microalgae as feedstock for production of char materials is its high water content (80–90%)^13^, which means that drying requires significant amounts of energy^14^. The need for drying can be eliminated by performing hydrothermal carbonization (HTC)^15,16^ to convert the microalgae into char materials known as hydrochars. HTC is performed in subcritical water at 180–300 °C, under autogenic pressure^17–19^, and is therefore suitable for wet feedstocks. Although the hydrochar retained a lower surface area than from pyrolysis biochars, its adsorption capacity can be higher than pyrochar due to its ion-exchange capacity and complexation^20^. HTC causes the feedstock to undergo various chemical transformations that increase its carbon density and reduce its H:C and O:C ratios, including decarboxylation, dehydration, demethanation, polymerization, and aromatization reactions^21,22^. Increasing the carbonization temperature and/or residence time increases the degree of carbonization of the final solid product, but the composition of the biomass—i.e. its content of carbohydrates (cellulose, hemicellulose, starch, sugars), lignin, lipids, proteins, and inorganics—affects both the minimum temperature required to initiate the HTC conversion^21,23,24^ and the physicochemical properties of the product^23,25^. Process variables such as pH can also affect the properties of the hydrochar; for example, adding acid enhances dehydration and thus significantly reduces the product’s oxygen content^26^. Hydrochars can also exhibit extensive surface functionalization^27,28^, making them potentially useful materials for removing heavy metals^6^, nutrients^29^, or dyes^30^ from water. Water pollution is a global concern^31–33^, that will require several different management methods. Hydrochars produced from waste streams have shown great potential for water remediation^34,35^, which may offer a possible valorization step for wet waste materials.

The process conditions found to affect the physicochemical properties of hydrochars include temperature, residence time, pH, feedstock composition, and pressure. These conditions affect the surface area, pore volume, pore size distribution, and functional groups such as carboxylic, phenolic, and hydroxyl groups on the hydrochar surface, known to govern the sorption properties^36^.

The conversion of wastewater-cultivated microalgae into valorised materials for water treatment and subsequent energy recovery would constitute an elegant material loop closure. Several studies have demonstrated the potential for using microalgae hydrochar in environmental applications^37–39^, but performance evaluation of un-activated microalgae hydrochar for removal of contaminants of emerging concern (CEC) or even dyes (e.g., methylene blue) has not been performed. CEC (e.g., pharmaceuticals) analysis is often both costly and time-consuming, so the adsorption capacity of carbonaceous sorbents is commonly evaluated using easily detected substances such as methylene blue, iodine, or molasses^40^.

The objectives of this study were to prepare hydrochars using native Swedish wastewater-cultivated microalgae as feedstock and to (i) evaluate the influence of HTC processing conditions (temperature, time, and pH) on the chemical composition and physicochemical properties of the resulting hydrochars, and (ii) assess the performance of the microalgae hydrochars as adsorbents using methylene blue. The study is, to our knowledge, the first to systematically examine the influence of time, temperature and pH on the microalgae hydrochar properties in connection to the adsorptive performance with regard to the methylene blue dye.

## Results and discussion

### Physicochemical characterization and DoE model responses

**Figure 1:**
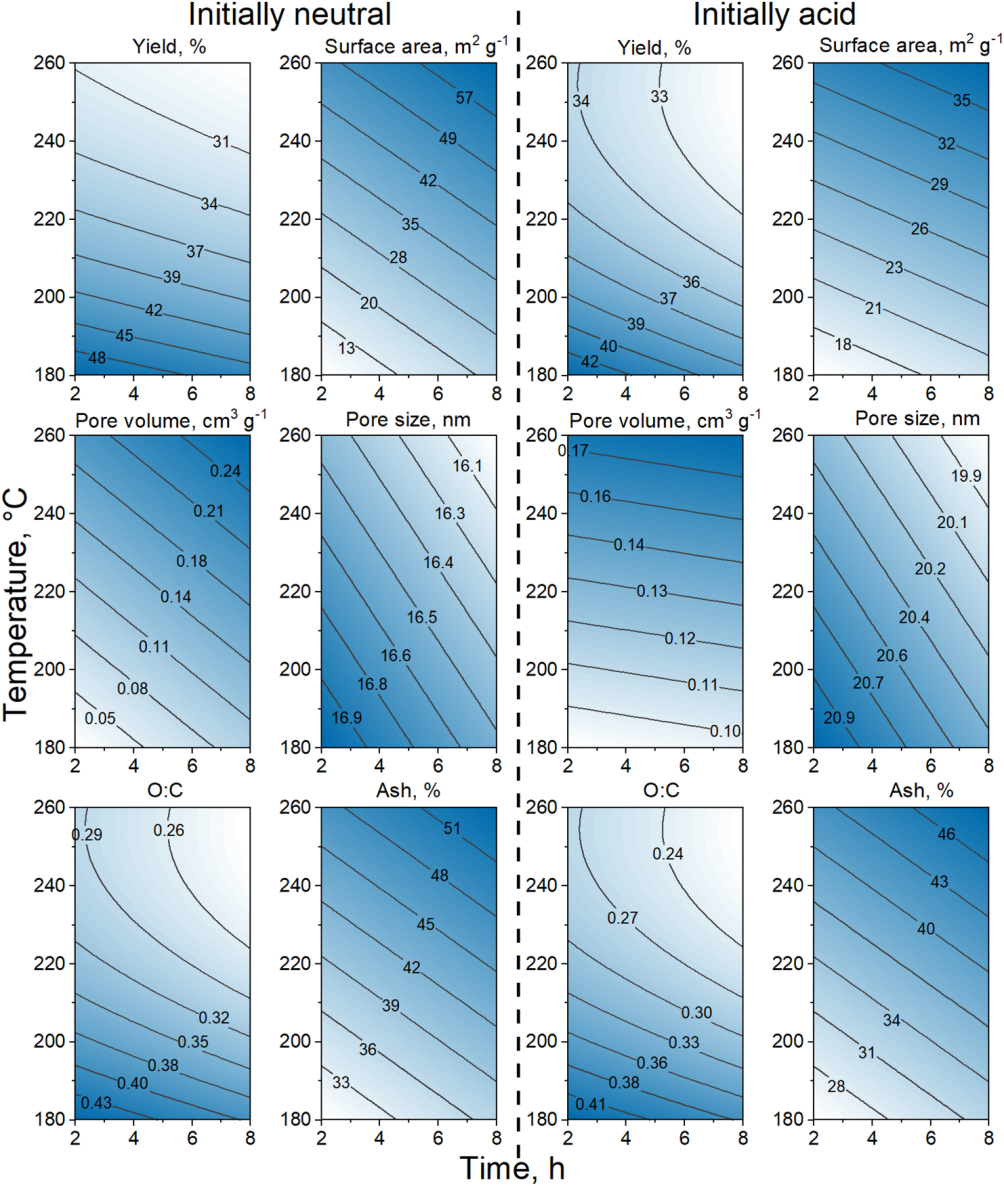
Surface plots for selected responses vs. carbonization temperature and residence time under initially neutral (left) and initially acidic (right) HTC processing conditions.

Individual response models were constructed based on the analyses of feedstock and hydrochar properties to assess the impact of carbonization temperature, residence time, and initial pH. The chosen responses were the solid yield (% dw), O:C atomic ratio, ash content (% dw), specific surface area (m^2^ g^−1^), pore volume (cm^3^ g^−1^), and average pore size (nm) of the hydrochars. To facilitate interpretation, the D-optimal design model responses were visualized using surface plots (Fig. 1), with darker colours corresponding to higher response values. As the plots in Fig. 1 show, HTC processing at 260 °C and 8 h resulted in an increased ash content, pore volume and surface area, and a reduced solid yield and N content (Supplementary Table S1, Supporting Information) compared to processing at lower temperatures and shorter residence times. For the solid yield and O:C ratio, the presence of quadratic terms in the regression models resulted in nonlinear response surfaces. A similar nonlinear influence of temperature on solid yield was observed by Álvarez-Murillo et al.^41^ in a study on carbonization of olive stones. The complete data set is provided in Supplementary Table S1, Supporting Information.

Solid yields varied between 29 and 53%, with low yields obtained at high carbonization temperatures and longer residence times (Fig. 1; Supplementary Table S1). The solid yields were lower than those typically obtained during HTC processing of lignocellulosic biomass (50–80% under comparable conditions)^21,23,24^ but similar to the solid yield of 14% obtained by Ekpo et al.^42^ after processing *Chlorella vulgaris* at 250 °C for 1 h. Microalgae and woody biomass have profoundly different chemical compositions and structures, which will likely affect the solid yield after HTC. Microalgae typically have higher protein and lipid content, whereas woody biomass is mainly composed of lignocellulosic materials^43^. The protein and lipid content of microalgae can undergo denaturation and decomposition during HTC, leading to higher conversion of the feedstock to liquid and gaseous products, and consequently a lower solid yield^44^. In woody biomass on the other hand, the biopolymers can undergo depolymerization and condensation reactions during HTC, leading to higher solid yield^45^.

The H:C ratio decreased from 1.6 in the feedstock to 1.3 after HTC at 260 °C for 8 h under initially acidic conditions, and the O:C ratio correspondingly changed from 0.5 to 0.2 (Fig. 1; Supplementary Fig. S1; Supplementary Table S1). H:C ratios around 0.3 have been associated with highly condensed aromatic structures, whereas H:C ratios ≥ 0.7 indicate no condensed aromatic structures^46–48^. When comparing the H:C and O:C ratios using the graphical methodology of van Krevelen^49^, the properties of hydrochars obtained at 180 °C closely resembled those of biomass and peat, while harsher conditions (i.e. higher temperature and longer residence time) yielded hydrochars more similar to lignite, albeit with less condensed aromatic structure (Supplementary Fig. S1)^46–48^. This observation suggests that HTC generates more condensed aromatic (i.e. lower H/C ratio), and more carbonised (lower O/C ratio) structures^48^.

Harsher reaction conditions increased the ash content of the hydrochars to between 23 and 53% dw, due to the decomposition of organic components and retention of inorganic matter (Fig. 1; Supplementary Table S1). Initial acidification of the reaction media reduced the ash content by increasing the solubility of alkali and alkaline earth metal salts, oxides and hydroxides in the liquid phase^50,51^. Higher carbonization temperatures also increased the specific surface area, the pore volume, and the pore size, which ranged from 8 to 68 m^2^ g^−1^, 0.03 to 0.29 cm^3^ g^−1^, and 15–25 nm, respectively (Fig. 1; Supplementary Table S1). The increase in temperature during HTC leads to more extensive carbonization reactions, and in turn to formation of a higher degree of carbonaceous structures. This can generate more micropores and mesopores, which will contribute to increased surface area and pore volume^52,53^. At higher temperatures, the hydrolysis and dehydration reactions occur more rapidly, which can lead to release of more volatile organic compounds and gases. A consequence of this is that more void spaces are created within the carbonaceous structures, resulting in an increase in pore size^54^. Higher HTC processing temperatures can also cause the water molecules to become more reactive, which accelerates hydrolysis and condensation reactions of organic molecules. This can promote formation of a more ordered and crystalline structure in the carbon materials, and contributing to the increase in surface area and pore volume^55^.

### Statistical evaluation of the DoE model

**Figure 2:**
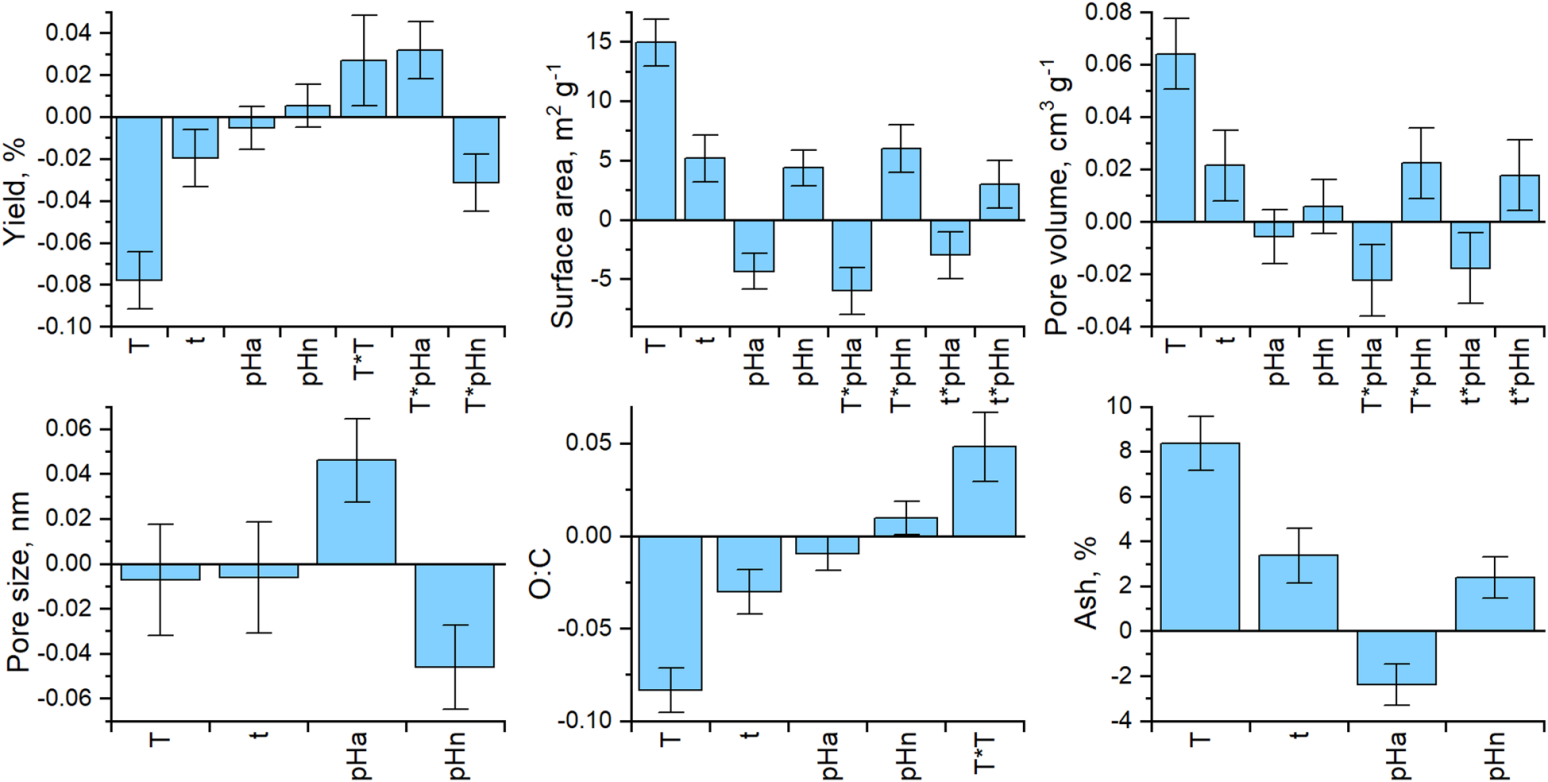
Selected regression model coefficients, with error bars indicating 95% confidence intervals. *T* Temperature, *t* time, *pH*_*a*_ initially acidic conditions, *pH*_*n*_ initially neutral conditions.

Evaluations of the statistical significance of the individual response models using ANOVA showed that the models fit the experimental data well in terms of predictive ability (*Q2* > 0.1), variability (*R2* > 0.5), and probability (*p* < 0.05) (Supplementary Table S2). The regression model coefficients (95% confidence level) (Fig. 2) indicated that carbonization temperature, residence time and initial pH had a statistically significant effect on the hydrochar’s properties. Temperature had the greatest effect on all responses other than pore size, which was mainly governed by the initial pH. Initially acidic conditions generated materials with lower pore volumes but greater pore sizes than initially neutral conditions. Residence time had a similar effect to temperature but with less pronounced impact on the studied responses, since the impact of time on the carbonization process is less direct than the temperature factor. Temperature is the main parameter affecting the shift of the chemical equilibrium towards formation of carbonaceous structures, as well as the extent of hydrolysis and dehydration reactions. The residence time can also have an effect on the extent of hydrolysis and dehydration reactions, but its impact on the degree of carbonization is less pronounced^23^. The statistical significance of the pore size model was significant but its predictive ability was not good (0.1 < *Q2* < 0.5)^56^. Only initial pH (acidic or neutral) was a significant factor for hydrochar pore size. Modelling based only on the initial pH as a qualitative factor, as defined in the DoE, was clearly insufficient for reliable prediction of hydrochar properties. It is worth noting that feedstock decomposition during HTC treatment generates acidity, reducing the pH of the reacting mixture^21^ explain why the initial pH had only a limited impact on the physicochemical properties of the hydrochar.

### DRIFTS analysis of microalgae hydrochars

**Figure 3:**
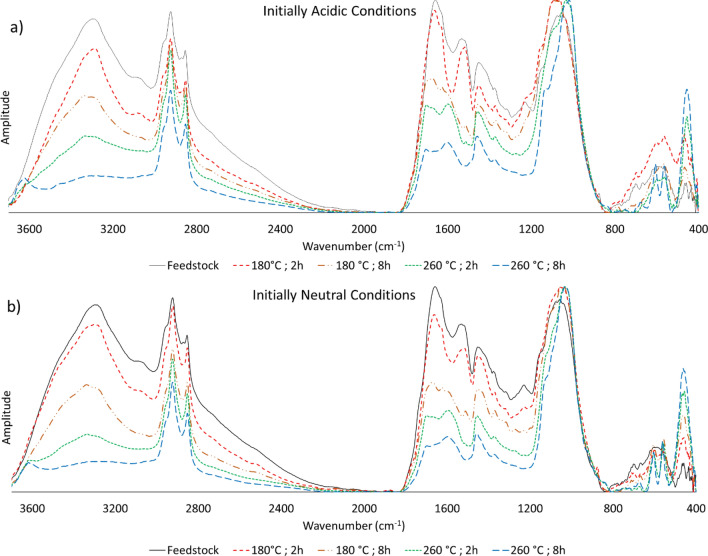
DRIFTS spectra of the microalgae hydrochars at 180 °C and 260 °C after 2 h and 8 h under (**a**) initially acidic conditions, and (**b**) initially neutral conditions.

The DRIFTS spectra show how the chemical structure and composition of the feedstock change as it is converted into hydrochar. HTC processing exhibited good overall reproducibility, especially under initially neutral conditions; the spectra of samples formed under such conditions were all very similar (Supplementary Fig. S2 (lower); Supporting Information), whereas those formed under initially acidic conditions displayed a higher degree of variation (Supplementary Fig. S2 (upper); Supporting Information). The spectra of hydrochars produced by heating at 180 °C for 2 h under both pH conditions were most similar to those of the initial microalgae biomass (Fig. 3), but increasing the residence time at the same carbonization temperature induced profound changes, especially in the 1700–1500 cm^−1^ range covering the protein amide 1 and 2 bands^57^. Bands in this range were less pronounced at 8 h compared to 2 h at 180 °C. This flattening of the protein amide bands is also apparent at 260 °C, indicating that the degree of protein breakdown increases at longer residence times and at higher temperatures, regardless of the initial pH of the feedstock slurry. C=C double bond stretches also appear in this region; in particular, aromatic ring stretching vibrations are typically centred in the region between 1510 and 1600 cm^−1^. The band shapes in this zone in the spectra of hydrochar prepared at high and low temperatures differ markedly, indicating that high temperature protein degradation is accompanied by an increase in the abundance of aromatic compounds. This is consistent with the fact that carbonization removes H and O, leading to a high proportion of unsaturated carbons. Under the tested experimental conditions, this led to the formation of aromatic compounds with carbon–carbon double bonds but not to the formation of alkynes (i.e., the spectra lack bands corresponding to triple bonds).

Another prominent feature is the weakening of the -OH bands in the 3700–3000 cm^−1^ range (attributed to OH groups of phenols, hydroxyls and carboxyls^57^), which became more pronounced at higher temperatures and longer residence times. Under the most extreme HTC conditions (260 °C, 8 h), a band appeared at ca. 3600 cm^−1^, indicating the presence of free OH groups or NH-functionalities^58^, This could be a new band arising, for example from the breakdown of proteins, or a previously obscured band revealed by the disappearance of the OH-band.

The aliphatic –C–H vibrations at 2926 cm^−1^, 2855 cm^−1^, and 1455 cm^−1^^59^, on the other hand, were largely unaffected by increasing the HTC temperature. Their apparent slight decrease in intensity is largely due to the disappearance of the overlapping bands (e.g., the –OH bands at high wavenumbers), which lowered their baseline.

The large group of (broad) bands covering the 1200–900 cm^−1^ region is dominated primarily by carbohydrate-related vibrations (ring breathing motions, glycosidic link stretches, etc.)^60^. The shape of this region of the spectra changed markedly with increasing HTC temperature and duration. In general, higher temperatures shifted these bands towards lower wavenumbers (i.e., lower energies), clearly indicating a change in carbohydrate composition. Under initially neutral conditions, this effect was observed even at lower temperatures (especially over longer times). In contrast, the carbohydrate bands remained at higher wavenumbers in hydrochars formed under initially acidic conditions at lower temperatures. Interestingly, the asymmetric –C–O–C– vibration (a shoulder at around 1120–1150 cm^−1^) became more pronounced at the highest temperatures (and even at lower temperatures under initially acidic conditions). The moiety responsible for this vibration is unknown, but it is likely to originate from (a) functionalization of carbohydrate hydroxyls (e.g., esterification, etherification), (b) linkages between monomer units in a polymeric structure, or (c) interlinkages between polymers. The fact that the –C–O–C– vibration becomes more pronounced does not necessarily indicate an increase in the prevalence of such functionalities; it can also (at least partly) be attributed to overlapping carbohydrate ring vibrations shifting to lower wavenumbers. It is known that HTC treatment generates acidic conditions as the breakdown of the organic structure proceeds^21^, but the observed spectral changes may indicate a reaction driven by the initially acidic conditions (c.f. Fig. 3a,b).

Another noteworthy detail of the DRIFTS spectra is the changes in the low fingerprint region. The broad, convoluted bands between 700 and 500 cm^−1^ are resolved into three distinct bands at carbonization temperatures above 220 °C (Fig. 3a,b). This could be due to inorganic components, as the bands at 606 and 560 cm^−1^ could originate from the P–O stretching vibration of PO_4_^3−^, and the band at 459 cm^−1^ from the Mg–O stretching vibration of the microalgal chlorophyll^61^. However, this region is also home to composite vibrations, so these bands could indicate the formation of a more uniformly structured hydrochar due to feedstock breakdown (e.g., the conversion of a complex mixture of carbohydrates into a more uniform set of breakdown products).

### Adsorption of methylene blue dye on microalgae hydrochars

**Figure 4:**
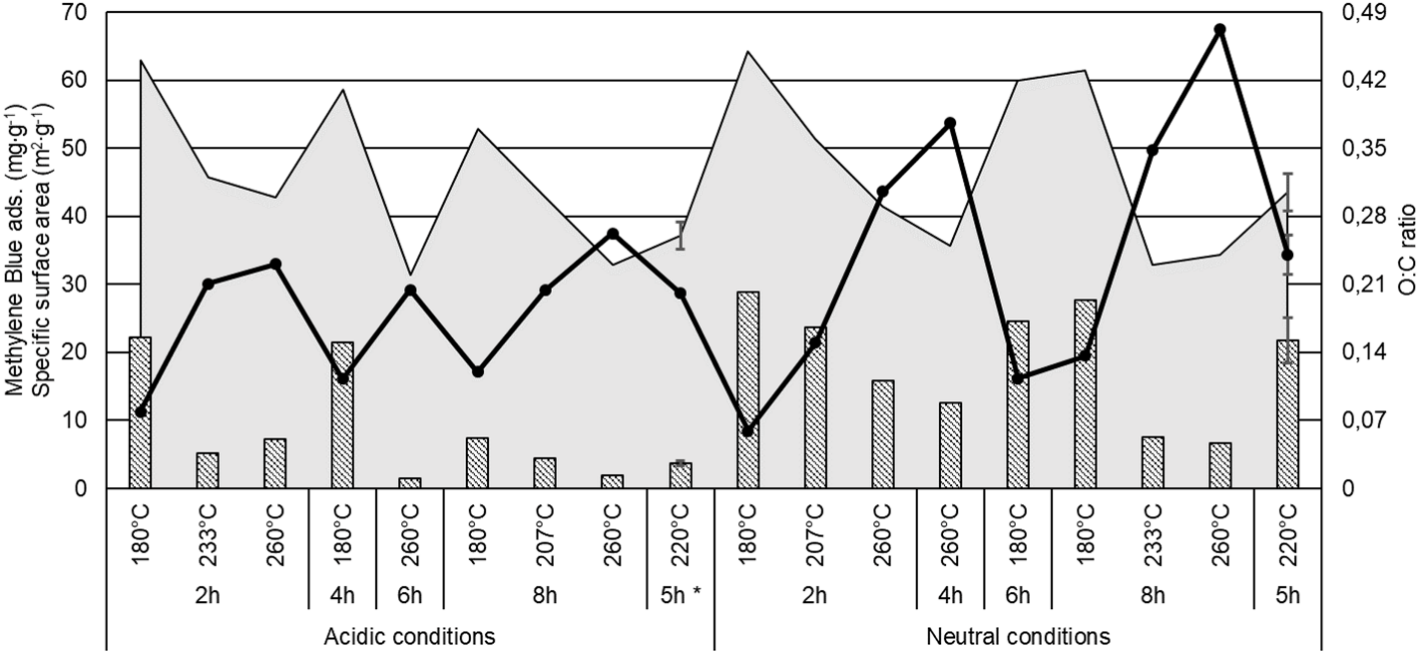
Adsorption of methylene blue (striped bars, scale on left axis) co-visualized with the specific surface area (black line, scale on left axis) and O:C ratio (solid grey area; scale on right axis) for all generated hydrochars. Values shown are single point values except those for 5 h at 220 °C, which are means of two samples in the initially acidic case and four samples in the initially neutral case. Error bars for initially acidic conditions represent the individual samples. Error bars for initially neutral conditions represent ± 1 standard deviation for the four centre point replicates.

Overall, methylene blue adsorption was higher for hydrochars obtained under initially neutral conditions than for those obtained under initially acidic conditions. The highest methylene blue adsorption capacity (28.9 mg g^−1^) was observed for hydrochars produced by HTC at 180 °C for 2 h under initially neutral conditions, while the lowest adsorption capacity (1.5–1.9 mg g^−1^) was observed for hydrochars produced at 260 °C for 6–8 h under initially acidic conditions (Fig. 4). The adsorption capacity was relatively high compared to e.g. 9.7 mg g^−1^ on rice husk hydrochar (260 °C; 1 h)^20^ and comparable with the 26.6 mg g^−1^ that optimized coffee husk hydrochar (210 °C; ~ 4 h)^62^ adsorbed but less than 40.0 mg g^−1^ obtained for citrus waste hydrochar^63^.

Both the pore volume and the specific surface area of the microalgae-derived hydrochars were generally low, with the highest observed values being 0.29 cm^3^ g^−1^ and 68 m^2^ g^−1^, respectively. For reference, the specific surface area of activated carbons typically ranges between 500 and 1500 m^2^ g^−1^, but can be as high as 3000 m^2^ g^−1^^64^, in accordance with the important contribution of surface interactions to their adsorptive capacity. The relatively low surface area of the hydrochars and the inverse correlation between their surface areas and their adsorption of methylene blue adsorption implies that adsorption is driven by a mechanism other than physical adsorption, e.g., π–π interactions, electrostatic interactions, or hydrogen bonding.

Hydrochars have been reported to be rich in oxygen-containing functional groups (e.g. carboxylic acids, lactones, and hydroxyls)^65,66^, which is consistent with our findings (Fig. 3). Methylene blue is a cationic dye that is positively charged in water. Consequently, negatively charged groups on the hydrochar surface may promote methylene blue adsorption through electrostatic attraction. Such a mechanism of adsorption of cationic dyes on hydrochars was proposed by Tran et al.^67^ to explain the efficient removal of methylene green dye (structurally very similar to methylene blue) by hydrochars derived from agricultural residues. The covariation of methylene blue adsorption, and the oxygen-containing surface functionalities displayed in Fig. 4 and discussed above, supports this proposed mechanism and indicates that the presence of oxygenated functional groups has a positive influence on the adsorption of methylene blue and presumably also that of other cationic compounds. Nevertheless, methylene blue adsorption was notably weaker for hydrochars produced under acidic conditions, even though their O:C ratios were similar to those of samples formed under neutral conditions. We hypothesize that this was because the excess protons present under acidic conditions reduced the number of negatively charged surface groups. As seen in Fig. 3, the initial pH strongly affected the carbohydrate composition (indicated by the bands seen in the 1200–900 cm^−1^ region of the DRIFTS spectra), showing that the addition of acid influences the functional groups in the hydrochar. As this difference is not visible at higher temperatures, there must be one or more parameters, e.g. microalgae feedstock composition, pressure, reactor configuration, presence of other cationic compounds, and/or hydrochar washing, which were not considered in this study but possibly at least partly influential in explaining the differences in methylene blue adsorption for high temperature hydrochars (Fig. 4). For this cationic dye, the effect of the initially acidic conditions on the hydrochar functional groups reduces the hydrochar’s ability to adsorb the dye. However, the opposite may be observed for anionic target compounds. By extension, these findings imply that hydrochar surface properties could be “tailored” by adjusting HTC processing conditions other than time and temperature^41^.

The systematic examination conducted within this study shows that carbonization temperature, residence time and initial pH all had statistically significant effects on microalgae hydrochars properties and their capacity for methylene blue adsorption. Carbonization temperature had the strongest effect on the hydrochar properties (i.e., specific surface area, pore volume, ash content, element composition and surface functionality) and DRIFTS spectra analysis revealed differences in the carbohydrate vibrational bands (1200–900 cm^−1^) between hydrochars produced under initially acidic conditions and those produced under initially neutral conditions at 180 °C. The effect on the carbohydrate functional groups seemed to be unrelated to residence time and were likely caused by acid—feedstock interactions. The effect was notable in the methylene blue adsorption assessment, as hydrochars produced at 180 °C for 8 h under initially neutral conditions adsorbed almost four times as much as hydrochar produced at the same temperature and residence time but under initially acidic conditions. Generally, hydrochars produced under initially acidic conditions had a lower capacity for methylene blue adsorption, suggesting that adsorption was driven mainly by electrostatic interactions with negatively charged oxygen groups on the surface of the microalgae hydrochars.

Overall, the study found that un-modified, wastewater grown, microalgae derived hydrochars could, despite their relatively low surface area, be used as adsorbents for the removal of organic substances from water by electrostatic interactions. This opens up a possible valorisation step for microalgae cultivated as part of a nutrient removal approach in municipal wastewater. Future work would involve using these findings regarding HTC treatment parameters to produce microalgae hydrochars with the aim to investigate their efficiency as adsorbents for CEC such as e.g. pharmaceuticals.

## Material and methods

### Microalgae biomass

A microalgae polyculture comprising green algae of the genera *Scenedesmus*, *Desmodesmus*, *Coelastrum* and *Chlorella* was provided by the Swedish University of Agricultural Sciences (Umeå, Sweden). In brief, the microalgae were grown in an open pond fed with municipal wastewater influent collected from the local wastewater treatment plant (Vakin, Umeå, Sweden) and flue gases from the local combined heat and power (CHP) plant (Umeå Energi, Umeå) as a carbon source and for pH regulation. Temperature and light were not controlled and followed natural variation. Additional details of the cultivation conditions are given in Lage et al.^68^. Microalgae were harvested once a week by sedimentation for about two days followed by continuous centrifugation at 5000 rpm (US Filtermaxx, Jacksonville, Florida, USA) to 15% wt. solids and were then stored in a freezer at − 20 °C until use^68^.

### Hydrothermal carbonization of microalgae biomass

Prior to HTC treatment, the frozen microalgae biomass was slowly thawed overnight. For each experiment, 10 g of homogenized (15 wt% solids) slurry and 8 mL of deionized water (the dry solid-to-water ratio was 1:12) were placed in a 25 mL polytetrafluoroethylene lined stainless steel autoclave reactor (Toption Lab, China). For experiments conducted under initially acidic conditions, 3 mL of the deionized water was replaced with 3 mL of 0.5 M hydrochloric acid (analytical grade). The obtained pH was approximately 2. After loading, the reactors were heated in a muffle furnace to the target temperature at an average heating rate of 3 °C min^−1^, after which the temperature was held for the target residence time, and then the reactors were cooled to room temperature before being depressurized and opened. Target temperatures and times are presented in Table 1. Solid and liquid products were separated by filtering the slurry using a 0.45 µm PTFE filter (Whatman, Fisher Scientific). The filter cake of the obtained hydrochars was washed with 100 mL of deionized water, followed by 20 mL of acetone (analytical grade), and lastly 100 mL deionized water to remove residual liquid products physisorbed on the surface. The solid product was oven-dried at 105 °C overnight, cooled, and weighted immediately to calculate yield.

**Table 1:** The D-optimal design, excluding replicates. The four centre point replicates were under initially neutral conditions, 220 °C, 5 h; and there were two additional replicates under initially acidic conditions, 220 °C, 5 h.

Exp. no.	1	2	3	4	5	6	7	8	9	10	11	12	13	14	15	16
Temp., °C	180	260	180	260	180	260	233	207	180	260	180	260	180	260	207	233
Time, h	2	2	8	8	4	6	2	8	2	2	8	8	6	4	2	8
pH	2	2	2	2	2	2	2	2	7	7	7	7	7	7	7	7

### Design of experiments (DoE)

A D-optimal design^56^ with replicated centre points was used to systematically examine the influence of carbonization temperature (180–260 °C; quantitative factor), residence time (2–8 h; quantitative factor), and initially neutral or acidic media (initial pH 7 and 2; qualitative factor) on solid yield and properties. The specific responses were solid yield, specific surface area, pore volume, pore size, nitrogen content, H:C ratio, O:C ratio, and total ash content. The D-optimal design (Table 1) comprised 22 individual experiments, including four replicates at neutral pH, run at the central point of the design (220 °C, 5 h) and two additional replicates at acidic pH (220 °C, 5 h).

The model was assessed using surface plots, which facilitate data evaluation by visualizing the functional relationships between a designated dependent response (e.g., yield), and two independent variables (i.e., temperature and time). Important terms in the D-optimal design model were identified using analysis of variance (ANOVA) and regression model significance was evaluated based on Fischer test probability (*p*) values, where *p* < 0.05 was considered to indicate model significance. The MODDE Pro software package (version 12.0, Umetrics AB, Sweden) was used for DoE planning and data evaluation.

### Analytical methods

#### Elemental composition

Elemental analysis was performed on the microalgae feedstock and the hydrochars using a CHNS-O elemental analyser model EA3000 (Eurovector Srl, Italy) according to DIN 51732^69^. Ash content was calculated by subtracting the CHNS-O content from the total weight.

#### Porosity properties

For the porosity analysis of the hydrochars, a TriStar 3000 automated nitrogen sorption/desorption instrument (Micromeritics, Norcross, GA, USA) was used. Before analysis, 0.2 g of dried hydrochar was degassed under nitrogen flow at 120 °C for 2 h using a Micromeritics Smart Prep degassing unit. The isotherms obtained for nitrogen adsorption–desorption on microalgae hydrochars were evaluated by the multipoint Brunauer–Emmett–Teller (BET) method to calculate the total specific surface area^70^. Pore volume was determined by the Barrett-Joyner-Halenda (BJH) method^71^.

#### Adsorption performance

The methylene blue adsorption capacity of the prepared hydrochars was determined at ambient temperature (20 °C) using the single-point adsorption method proposed by Raposo et al., (2009)^40^. In brief, 50 mg hydrochar and 50 mL methylene blue solution (50 mg L^−1^) were combined in closed 50-mL tubes and shaken by an orbital shaker at 50 rpm for 8 h, after which the solution was filtered off using a 0.45 µm nitrocellulose membrane syringe filter (Filtropur, Sarstedt). Preliminary lab tests, supported by published methods^30,72,73^, confirmed that 8 h was sufficient to reach adsorption equilibrium in the solution. Filtrate methylene blue concentration was determined with a Helios 8 UV/VIS spectrophotometer at 668 nm using a 1 cm quartz cuvette. When required, the samples were diluted to the concentration range 1–5 mg L^−1^. The equilibrium adsorption (*q*_*eq*_) was calculated as follows:

1 $${q}_{eq}=\frac{\left({C}_{0}-{C}_{eq}\right)\cdot V}{m},$$

where *C*_0_ (initial) and *C*_*eq*_ (equilibrium) are the concentrations of methylene blue (mg L^−1^), *V* is the volume of methylene blue solution (L), and *m* is the mass of sorbent (g).

#### Surface functionalities

The chemical compositional changes of the hydrochars were analysed by Diffuse Reflectance Infrared Fourier Transform Spectroscopy (DRIFTS) using a Bruker IFS 66 v/S FT-IR spectrometer with a standard DTGS detector. Prior to analysis, the hydrochars were ground with KBr at a ratio of ca. 1:12. Measurements were performed under vacuum conditions (below 0.7 kPa), covering the spectral range 4000–400 cm^−1^ at 2 cm^−1^ resolution. Spectra were baseline corrected using a linear technique with automatic points selection and min–max normalization over the entire spectral range with the KnowItAll software (John Wiley & Sons).

## Supplementary Information

Supplementary Information.

## Data Availability

All relevant data generated or analysed during this study are included in this published article and in the supplementary information. Additional parts of the datasets used and/or analysed during the current study are available from the corresponding author upon request.
