# Supplementary material for: Influence of hydrothermal carbonization conditions on the porosity, functionality, and sorption properties of microalgae hydrochars
Source: Sci Rep. 2023 May 26;13:8562. doi: 10.1038/s41598-023-35331-0 (PMC10219968; doi:10.1038/s41598-023-35331-0)
Supplement: Supplementary file 1 — Supplementary Information. [file 41598_2023_35331_MOESM1_ESM.pdf]

Supplementary information

Influence of hydrothermal carbonization conditions on the porosity, functionality, and sorption properties of microalgae hydrochars

Table S1. The experimental design and properties of hydrochars obtained by hydrothermal carbonization of algae biomass

	Experimental conditions (design)			Hydrochars' properties											
Sample	Temperature, °C	Time, hours	pH	Yield,	Specific surface area, $m^2\ g^{-1}$	Pore volume, $m^2\ g^{-1}$	Pore size, nm	C, wt%	H, wt%	N, wt%	O, wt%	Total ash, wt%	H:C	O:C	MB adsorption, $mg\ g^{-1}$
Feedstock	-	-	-	-	-	-	-	42.9	5.7	5.8	26.6	17.9	1.58	0.47	-
1	180	2	2	42.8	11.3	0.06	22.7	41.2	5.4	5.4	24.2	22.9	1.56	0.44	22.2
2	260	2	2	34.1	33.0	0.17	21.0	37.6	4.4	2.9	15.0	39.2	1.39	0.30	7.3
3	180	8	2	37.1	17.1	0.08	19.0	40.8	5.0	3.2	20.0	30.1	1.46	0.37	7.4
4	260	8	2	32.6	37.5	0.16	17.4	34.6	3.7	2.5	10.5	47.7	1.27	0.23	1.9
5	180	4	2	41.2	16.1	0.08	20.1	41.4	5.0	4.0	22.5	26.1	1.44	0.41	21.5
6	260	6	2	31.4	29.2	0.15	20.2	35.3	4.0	2.5	10.5	46.7	1.35	0.22	1.5
7	233	2	2	33.1	30.1	0.14	18.4	37.9	4.3	2.8	16.0	38.1	1.35	0.32	5.2
8	207	8	2	36.9	29.1	0.13	18.2	38.6	4.3	2.8	15.3	38.0	1.33	0.30	4.5
9	180	2	7	52.8	8.5	0.03	16.3	37.5	5.0	3.4	22.7	30.5	1.59	0.45	28.9
10	260	2	7	30.7	43.6	0.17	15.5	34.3	4.0	2.6	13.3	44.9	1.39	0.29	15.9
11	180	8	7	47.8	19.5	0.08	16.4	36.2	4.4	2.6	20.8	35.0	1.45	0.43	27.7
12	260	8	7	28.9	67.5	0.29	17.3	30.5	3.5	2.3	9.6	53.0	1.37	0.24	6.6
13	180	6	7	46.5	16.2	0.07	17.6	35.5	4.4	2.8	20.0	36.3	1.48	0.42	24.6
14	260	4	7	31.0	53.7	0.22	16.4	32.4	3.7	2.6	10.7	49.6	1.36	0.25	12.6
15	207	2	7	41.5	21.4	0.08	15.8	37.9	4.6	2.8	18.1	35.6	1.45	0.36	23.7
16	233	8	7	28.7	49.8	0.21	16.9	34.9	3.9	2.8	10.6	46.7	1.33	0.23	7.5
17	220	5	7	35.0	36.0	0.13	14.9	35.2	4.0	2.7	13.7	43.4	1.35	0.29	24.3
18	220	5	7	37.1	30.9	0.12	15.2	36.3	4.2	2.7	15.9	39.9	1.38	0.33	18.0
19	220	5	7	32.6	37.4	0.17	17.8	34.9	4.0	2.6	13.3	44.1	1.37	0.29	24.9
20	220	5	7	36.2	33.1	0.15	18.1	35.5	4.1	2.6	14.8	42.0	1.38	0.31	19.9
21	220	5	2	35.5	28.4	0.18	25.1	37.6	4.1	2.9	12.5	41.9	1.30	0.25	3.7
22	220	5	2	37.2	29.0	0.17	23.1	38.7	4.2	3.1	13.9	39.1	1.29	0.27	3.0
Average Initially Acidic				36,2 ± 3,7	26,1 ± 8,3	0,13 ± 0,04	20,5 ± 2,5	38,4 ± 2,3	4,4 ± 0,5	3,2 ± 0,9	16 ± 4,8	37 ± 8,2	1,37 ± 0,09	0,31 ± 0,07	7,8 ± 7,7
Average Initially Neutral				37,4 ± 8	34,8 ± 17,1	0,14 ± 0,07	16,5 ± 1	35,1 ± 2	4,2 ± 0,4	2,7 ± 0,3	15,3 ± 4,3	41,8 ± 6,6	1,41 ± 0,07	0,32 ± 0,08	19,6 ± 7,5

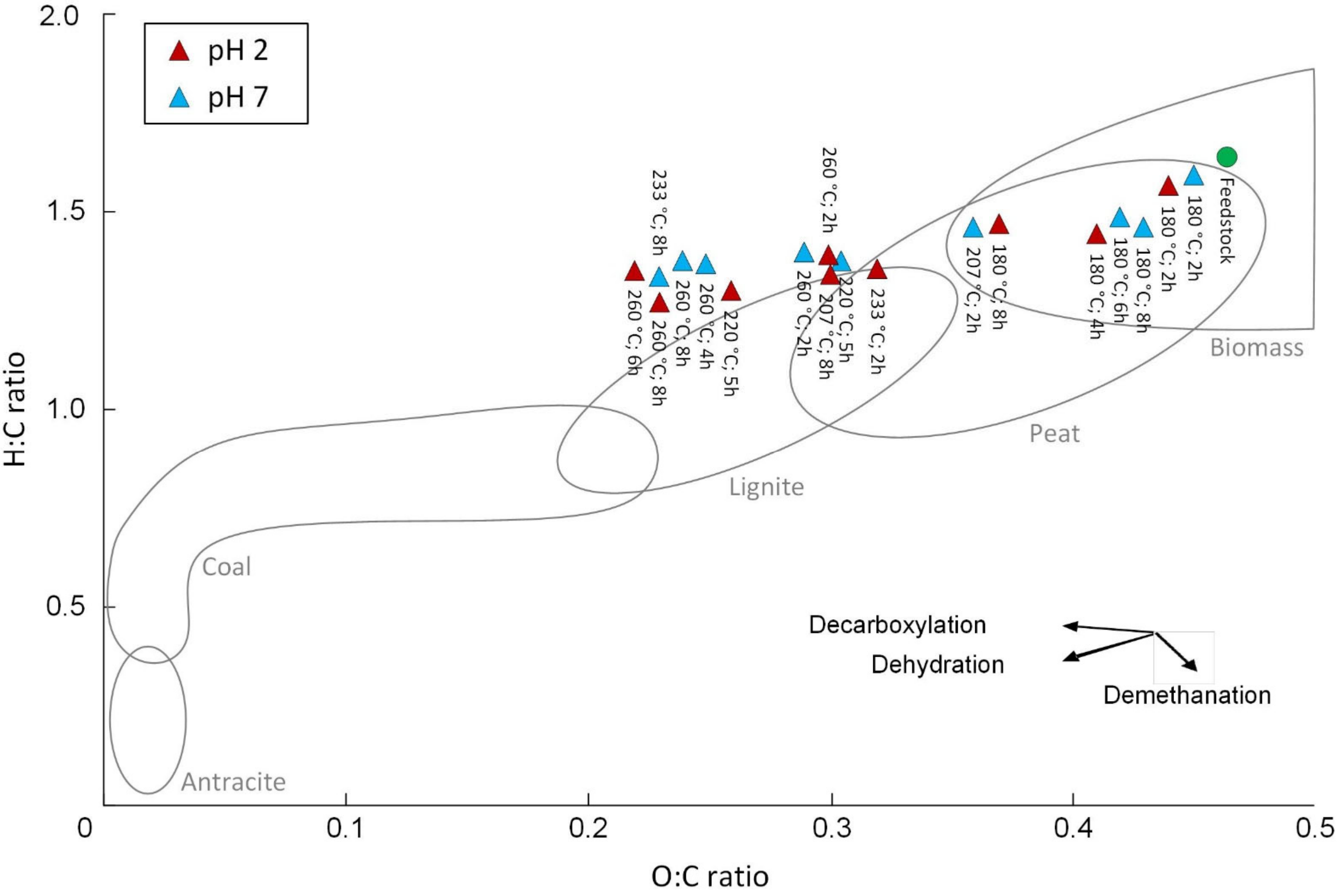

Figure S1. Van Krevelen diagram for H:C and O:C ratios of microalgae biomass and hydrochars. Red triangles denote initially acidic conditions, blue triangles initially neutral conditions.

Table S2. The statistical significance of process variables for individual response models

Responses	Statistical parameters (criteria for model significance)		
	Predictive ability, *Q2* (>0.1)	Variability, *R2* (>0.5)	Probability, *p* (<0.05)
Solid yield	0.862	0.922	$2.6\ 10^{-8}$
Specific surface area	0.915	0.959	$1.6\ 10^{-10}$
Pore volume	0.805	0.892	$3.3\ 10^{-7}$
Pore size	0.440	0.606	$6.5\ 10^{-4}$
Total ash	0.910	0.938	$4.9\ 10^{-11}$
H:C	0.803	0.892	$4.2\ 10^{-7}$
O:C	0.904	0.943	$2.5\ 10^{-10}$
Methylene blue adsorption	0.784	0.856	$8.8\ 10^{-8}$

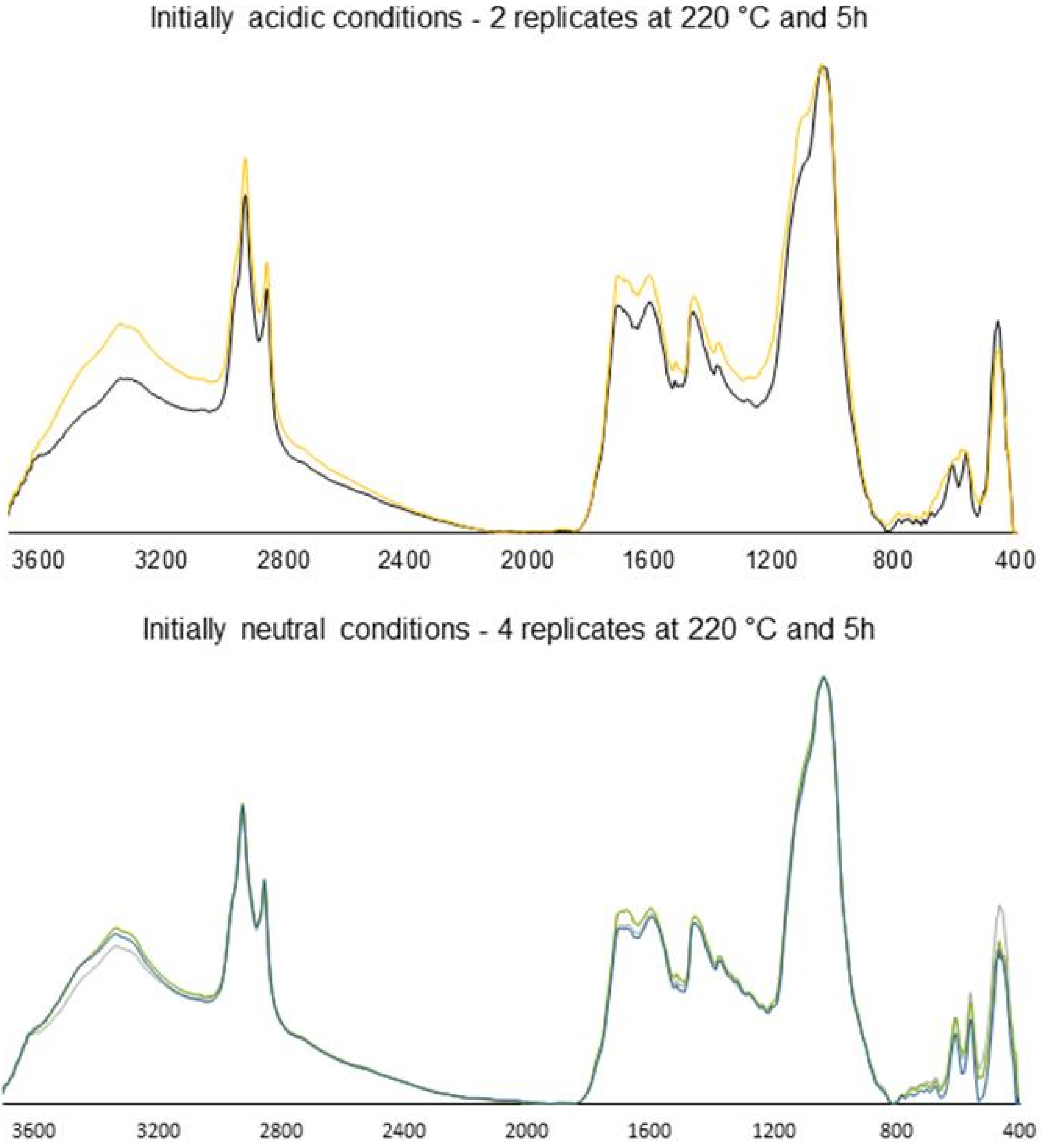

Figure S2. DRIFTS spectra of the DoE centre points run.
